# Ubiquitous expressed transcript promotes tumorigenesis by acting as a positive modulator of the polycomb repressive complex 2 in clear cell renal cell carcinoma

**DOI:** 10.1186/s12885-019-6069-3

**Published:** 2019-09-03

**Authors:** Jin Zeng, Wei Xiang, Yucong Zhang, Chunhua Huang, Ke Chen, Zhiqiang Chen

**Affiliations:** 1 Department of Urology, Tongji Hospital, Tongji Medical College, Huazhong University of Science and Technology, Wuhan, 430030 People’s Republic of China; 2 Department of Urology, the First Affiliated Hospital of Nanchang University, Nanchang, 330000 People’s Republic of China; 3 College of Basic Medicine, Hubei University of Traditional Chinese Medicine, Wuhan, 430065 People’s Republic of China; 4 Department of Geriatric, Tongji Hospital, Tongji Medical College, Huazhong University of Science and Technology, Wuhan, 430030 People’s Republic of China

**Keywords:** UXT, EZH2, PRC2, HMTase activity, ccRCC, Cell migration, Cell invasion

## Abstract

**Background:**

The ubiquitous expressed transcript (UXT) plays a key role in various tumors by regulating transcriptional activity of multiple transcription factors, including androgen receptor (AR). However, the role of UXT in clear cell renal cell carcinoma (ccRCC) is still unknown.

**Methods:**

Yeast two-hybrid screening, GST pull-down and co-immunoprecipitation assays were performed to detect the interacting protein of UXT. Chromatin immunoprecipitation (ChIP) was performed to investigate the levels of histone H3 lysine 27 trimethylation at the HOXA9 promoters. CCK-8 assays, colony formation assays and Transwell assays were performed to detect the proliferation, colony formation, migration and invasion of renal cancer cells. Quantitative PCR analysis was performed to detect the expressions of UXT in human ccRCC samples.

**Results:**

The enhancer of zeste homolog 2 (EZH2) is a novel UXT interacting protein and UXT interacts with EZH2 in the nucleus. In addition, UXT interacts with the polycomb repressive complex 2 (PRC2) through directly binding to EZH2 and suppressor of zeste 12 homolog (SUZ12), but not to embryonic ectoderm development (EED). Moreover, the UXT activates EZH2 histone methyltransferase activity by facilitating EZH2 binding with SUZ12. We further provided striking evidences that knockdown of UXT inhibits proliferation, colony formation, migration and invasion of renal cancer cells, in an EZH2-dependent manner. Importantly, the upregulation of UXT expression was observed in clinical ccRCC samples, and the high expression level of UXT was associated with advanced stage, distant metastasis and poor overall survival in patients with ccRCC.

**Conclusion:**

The UXT is a novel regulator of the PRC2 and acts as a renal cancer oncogene that affects the progression and survival of ccRCC patients.

**Electronic supplementary material:**

The online version of this article (10.1186/s12885-019-6069-3) contains supplementary material, which is available to authorized users.

## Background

Renal cell carcinoma (RCC) is a common urologic tumor, with 338,000 new cases and accounts for about 144,000 deaths worldwide in 2012 [1]. The most prevalent RCC is clear cell renal cell carcinoma (ccRCC) which accounts for about 70–80% of RCC [2]. Previous studies have identified many driver genes that associated with ccRCC, including *VHL* [3], *PBRM1* [4, 5], *BAP1* [6], *SETD2* [3, 7], *TCEB1* [3, 8], *KDM5C* [3, 9], *HIF1A* [10], *HIF2A* [11, 12] and *PTEN* [3]. In spite of extensive ongoing researches, mechanisms underlie the progression of ccRCC are still not fully understood.

The ubiquitous expressed transcript (UXT), also named as androgen receptor trapped clone 27 (ART27), is expressed widely in all tissues of both human and mouse [13]. The best-known function of UXT is regulating multiple transcription factors, including AR, GATA4, NF-κB, and EVI1 [14–18]. It was shown that UXT can regulate expressions of AR downstream genes as a co-factor, by interacting with VHL and URI/RMP [13, 19]. In addition, UXT regulates cell viability by interacting with centrosome [20]. Other investigations suggested UXT to be a component of the TNF receptor signaling complex, which plays a critical role in anti-viral pathway [21, 22]. Deregulations of AR, GATA4, NF-κB and EVI1 signaling pathways are key factors that contribute to the progression of various malignancies. Although UXT was found ubiquitously expressed in human tissues, UXT expression is upregulated in multiple tumor tissues, including colorectal cancer [23], sarcoma [18], and breast tumor [24]. Thus, UXT has been thought to be a pro-oncogene in human cancer. However, McGilvray et al. report that UXT suppresses EVI1-mediated cell transformation [17]. It has also been reported that UXT is downregulated in prostate cancer tissue and its over-expression inhibited the prostate cancer cell growth [25–27]. Thus, UXT may both promote and suppress tumorigenesis, depending on the tumor types and microenvironments.

In the present study, the enhancer of zeste homolog 2 (EZH2) was identified as a novel UXT interacting protein by using yeast two-hybrid screening (Y2H). Besides, we demonstrated that UXT interacts with the polycomb repressive complex 2 (PRC2) through directly binding to EZH2 and suppressor of zeste 12 homolog (SUZ12), but not to embryonic ectoderm development (EED). UXT promotes the formation of PRC2 complex and increases EZH2 histone methyltransferase (HMTase) activity, which subsequently inhibits the expression of many tumor suppressor genes such as *DAB2IP* and *HOXA9*. Our data also showed that the expression of UXT was upregulated in ccRCC tissues compared with paracarcinoma tissues. Moreover, in RCC cells, the knockdown of UXT resulted in the inhibition of cell proliferation, colony formation, and cell migration. We also found that the effect of UXT depletion on these three activities was reversed by concomitant EZH2 overexpression. Strikingly, we also found that overexpression of UXT correlates with advanced TNM stage, distant metastasis, and poor survival in patients with ccRCC. Collectively, our investigation reveals that UXT is a new regulator of the PRC2 complex and is essential for its tumor promotion function in ccRCC.

## Methods

### Reagents

Flag, HA, and Myc, EZH2, Lamin A/C, DAB2IP, HOXA9, Histone H3, H3K27me3 (histone H3 lysine 27 trimethylation), and GAPDH antibodies were previously described [28–31]; EZH2 knockout and wild-type 786-O cells were previously described [30]. Glutathione Sepharose 4B resin (17–0756-01) was purchased from GE Healthcare.

### Clinical tissue sample collection

Tumor tissues and normal adjacent tissues from pathologically and clinically confirmed ccRCC patients were kept in liquid nitrogen for total RNA extraction after radical nephrectomy. Written informed consent was obtained from all patients. This study was approved by the Tongji hospital of Tongji Medical College, Huazhong University of Science and Technology (Wuhan, China) ethics review committee.

### Constructs

The Y2H bait plasmid BD-UXT, mammalian expression plasmids for Flag- or HA- tagged EZH2, SUZ12, and EED were constructed as previously described [13, 30]. Mammalian expression plasmids for human Cherry-, EGFP-, Flag-, GST-, or HA-tagged EZH2 and UXT and its truncated mutants were constructed by standard molecular biology techniques. PCDH-H1, which contains a lentiviral vector backbone, was constructed by subcloning the H1 promoter of pSilencer5.1-H1 Retro (Invitrogen) into the ClaI and NotI sites of pCDH-CD513B-1 (System Biosciences). Gene-specific shRNA target sequence was synthesized, annealed and cloned into the BamHI and NotI sites of the PCDH-H1 plasmid. The primers for making these constructs were listed in Additional file 1: Table S1. All plasmids were verified by sequencing.

### RT-PCR, real-time PCR, and ChIP-qPCR analysis

Total RNAs extraction and cDNAs synthesis were performed according to the instructions of manufacturer using Trizol (Invitrogen) and ReverTra Ace qPCR RT Kit (TOYOBO), respectively. SYBR Green qPCR Master Mix (Roche) using ABI ViiA7 Real-time PCR System (Applied Biosystems) were used to perform real-time PCR. ChIP (chromatin immunoprecipitation) assays were performed as previously described to investigate the levels of H3K27me3 at the HOXA9 promoters [30]. Final analysis was performed using qPCR and shown as fold enrichment of the HOXA9 gene promoter.

### Y2H analysis

The detail of Y2H screening was described previously [13]. BD-­UXT was transformed into yeast strain AH109 as the bait. Then, these yeast colonies were collected and transformed with cDNA library (Clontech). Positive clones’ identification was performed by using SD-Leu-His-Ade selection plates and β-galactosidase assay. Positive plasmids were amplified in *Escherichia coli* DH5α and isolated by Endo-Free Plasmid Mini Kit II (Omega). BD-UXT or pGBKT7 control plasmid was co-transformed with these positive plasmids to confirm the specific interactions.

### GST pulldown and co-immunoprecipitation assays

GST pull-down assays was performed as previously described [32]. *Escherichia coli* cells were transformed with GST, GST–UXT, or GST-EZH2 plasmid and then lysed in NETN buffer. The GST recombinant proteins’ purification was performed using the glutathione-Sepharose 4B resin. Next, these proteins were incubated with the lysates from HEK293T cells, which were transfected with Flag­UXT or Flag-EZH2. Co-immunoprecipitation experiments were performed by using HEK293T cells or 786-O cells to analyze protein interactions [28].

### Cell proliferation, colony formation, cell migration, and invasion assays

After seeding 1000 cells per well in 6-well plates, colony formation assays were measured twelve days later. According to the manufacturer’s instructions, the CCK-8 (Dojindo Laboratories) was used to detect cell proliferation. Uncoated and Matrigel-coated Transwell inserts were used to perform cell migration and invasion assays. All experiments were performed in three independent experiments.

### Analysis of TCGA ccRCC samples

The TCGA data about mRNA (RNA Seq v2) expression levels in ccRCC patients were obtained from https://confluence.broadinstitute.org. Clinical data and recent follow-up data of ccRCC were downloaded from an integrated TCGA Pan-Cancer Clinical Data Resource [33]. In addition, samples lacking information about TNM, or grading and samples revealed the non-ccRCC phenotype were excluded from the analyses [34]. Differential expression between ccRCC and normal kidneys was calculated using the downloaded RSEM values. Survival curve was plotted using the Kaplan–Meier method and compared with the log-rank test.

### Statistical analysis

The data is presented as the means ±SD. Comparisons between two groups were performed using an unpaired Student’s t-test. Three levels of significance were used (*p < 0.05, **p < 0.01, and ***p < 0.001).

## Results

### Identification of EZH2 as a novel UXT binding protein

**Fig. 1:**
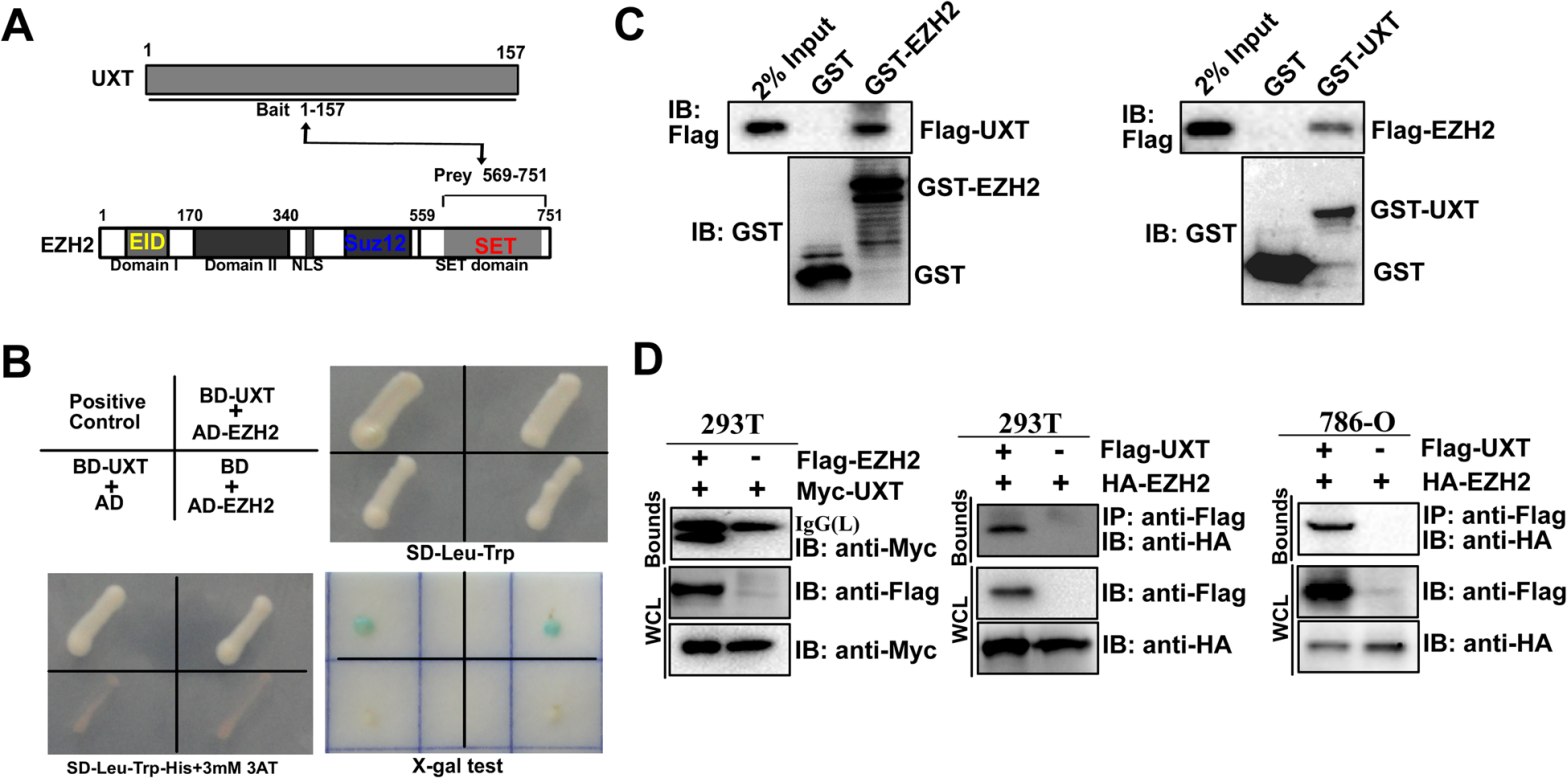
UXT interacts with EZH2. **a** Identification of the interaction between UXT and C­terminal fragment of EZH2 by the yeast two­hybrid approach. **b** EZH2 interacts with UXT in a yeast two­hybrid assay. Yeast AH109 cells were co­transformed with the indicated combinations of plasmids (left, top). The yeast colonies containing these plasmids were grown on SD-Leu-Trp (left, bottom) agar plates and on SD-Leu-Trp-His with 25 mM 3AT (3­Amimo­1,2,4­Triazole) agar plates (right, top) and were tested by the X­Gal assay (right, bottom). Abbreviations: AD, pGADT7; BD, pGBKT7. **c** In vitro GST pull-down assays. The GST, GST-EZH2, or GST-UXT purified from *E. coli* was incubated with Flag­UXT- or Flag-EZH2­expressing HEK293T lysates and precipitated with glutathione­Sepharose, respectively. Precipitates were subjected to SDS­PAGE and examined by immunoblotting with anti­Flag or anti-GST antibodies. **d** Co­IP of UXT and EZH2. Immunoprecipitation of Myc–UXT (left) and HA-EZH2 (middle and right) with anti-FLAG antibody, respectively. Cell extracts from 293 T cells (left and middle) or 786-O cells (right) with equal amounts of proteins were immunoprecipitated with an anti-FLAG antibody and analyzed by Western blotting using anti-Myc or anti-HA antibodies

UXT is associated with human tumorigenesis [20, 23]. However, the regulatory mechanisms associated with UXT-mediated tumorigenesis are still not well understood. To further investigate the downstream protein of UXT, a yeast two-hybrid screen was performed with UXT as bait protein. A total of 14 positive clones from 2 × 10^6^ transformants were identified (Additional file 2: Table S2). One of these clones encodes partial C-terminal region of EZH2 (Fig. 1a). To further verify the interaction between EZH2 and UXT, BD­UXT and AD­EZH2 were co-transformed into AH109 yeast cells (Fig. 1b, left, top). Reporter gene was found to be significantly activated after co-expression of UXT and EZH2 (Fig. 1b, bottom). To determine the direct interaction between UXT and EZH2, a GST pull-down assay was used. As expected, UXT protein was detected after incubated with GST–EZH2 but not GST, which indicated the specific binding between UXT and EZH2 (Fig. 1c, left). In a reciprocal assay, EZH2 was also detected to bind with GST-UXT (Fig. 1c, right). Further co-immunoprecipitation assays showed that Myc-UXT was co-precipitated efficiently with Flag-EZH2 and HA-EZH2 was also co-precipitated efficiently with Flag-UXT in 293 T cells (Fig. 1a).

### UXT associated with EZH2 in the nucleus

**Fig. 2:**
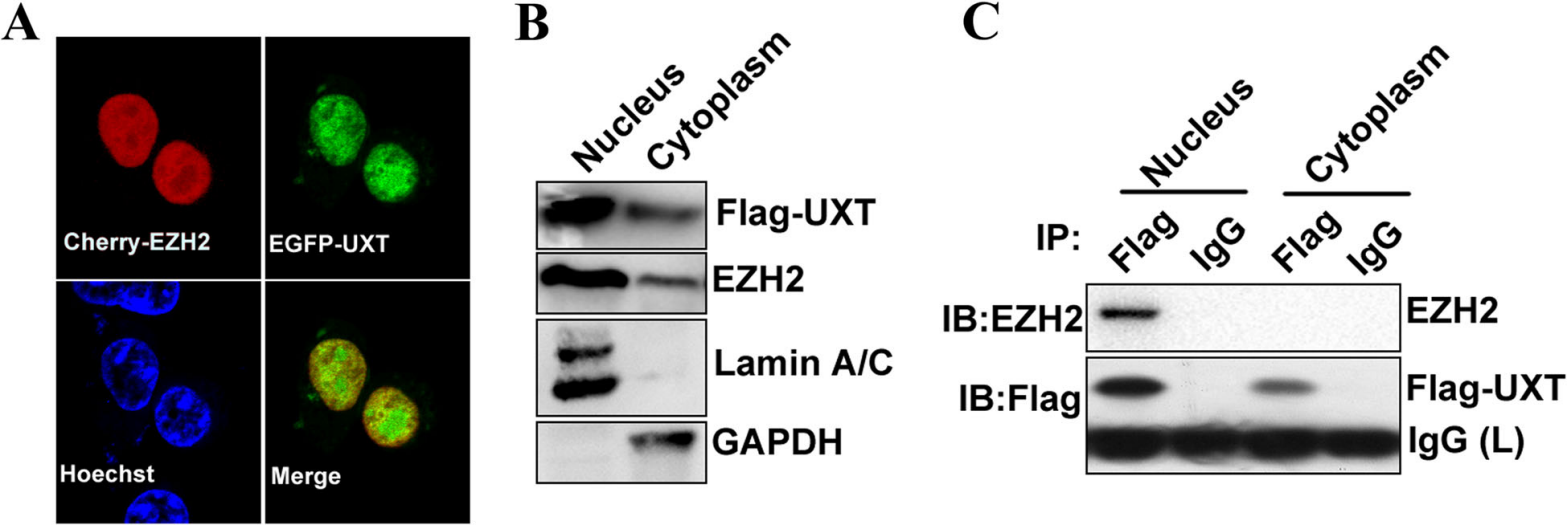
UXT interacts with EZH2 in the nucleus. **a** Confocal microscopy shows the co-localization of EZH2 with UXT. 293 T cells were transfected with the EGFP–UXT and mCherry–EZH2 plasmids. The nuclei were stained with Hoechst 33258. **b** Western blot analysis confirmed the predominant expression of UXT and EZH2 protein in the nucleus of 293 T cells. **c** Endogenous EZH2 interacts with UXT in the nucleus. FLAG–UXT was transfected into 293 T cells. The nuclear and cytoplasmic fractions were isolated. Equal amounts of the fractions were immunoprecipitated with either control IgG or anti-FLAG antibody, followed by probing with an anti-EZH2 antibody

For further investigation of intracellular colocalization and interaction of UXT and EZH2, EGFP-tagged UXT and mCherry-tagged EZH2 were co-expressed in 293 T cells by transfecting recombinant plasmids. Co-localization experiments showed that UXT and EZH2 were colocalized in the nucleus (Fig. 2a). Further cell fractionation and Western blot analysis showed that both Flag-UXT and endogenous EZH2 are predominantly expressed in the nucleus (Fig. 2b). In addition, immunoprecipitation assays showed that endogenous EZH2 was co-precipitated efficiently with Flag-UXT in nucleic nucleus but not in cytoplasmic components (Fig. 2c). Taken together, these results confirm that UXT interacts with EZH2 in the nucleus.

### Identification of the interacting domains of EZH2 with UXT

**Fig. 3:**
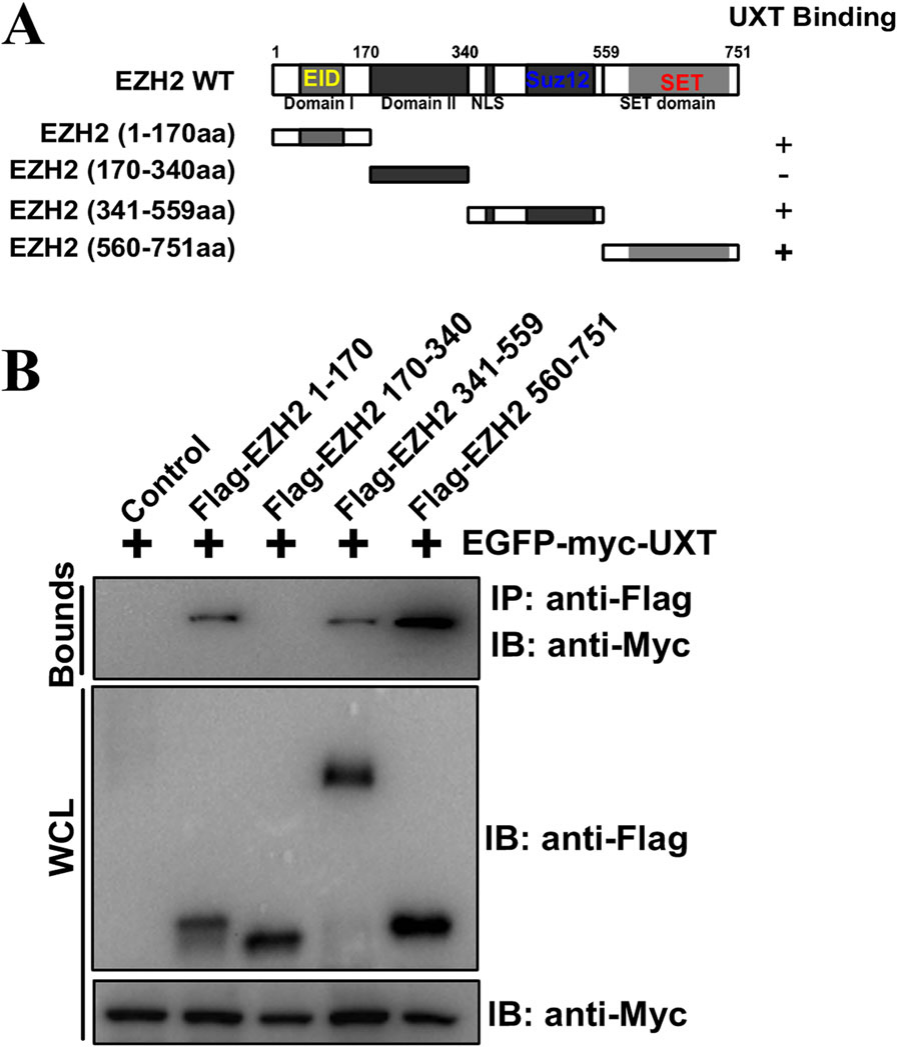
Identification of the EZH2 domains required for interaction with UXT. **a** Schematic representation of various deletion mutants of Flag-EZH2 used for co-immunoprecipitation analyses. **b** EGFP-myc-UXT was co-transfected with various Flag-EZH2 mutated plasmids and followed by Flag-immunoprecipitations and immunoblotting with Myc antibody

Next, we delineated the domains that respond for the interaction between UXT and EZH2 by co-expression of EGFP-myc-UXT and Flag-EZH2 deletion mutants. Considering UXT is a small protein and only contains one α domain [35], only UXT interacting domains of EZH2 were investigated. A series of EZH2 deletion mutants, which contain the Domain I [amino acids (aa) 1–170], Domain II [aa 170–340], SUZ12 binding domain [aa 341–559], and SET domain [aa 560–751], was co-expressed with EGFP-myc-UXT in HEK293T cells for co-immunoprecipitation assays (Fig. 3a). These results indicate that Domain II of EZH2 is insufficient to interact with UXT, but Domain I and SUZ12-binding domains showed decreased binding to UXT (Fig. 3b).

### UXT facilitates EZH2 binding with SUZ12

**Fig. 4:**
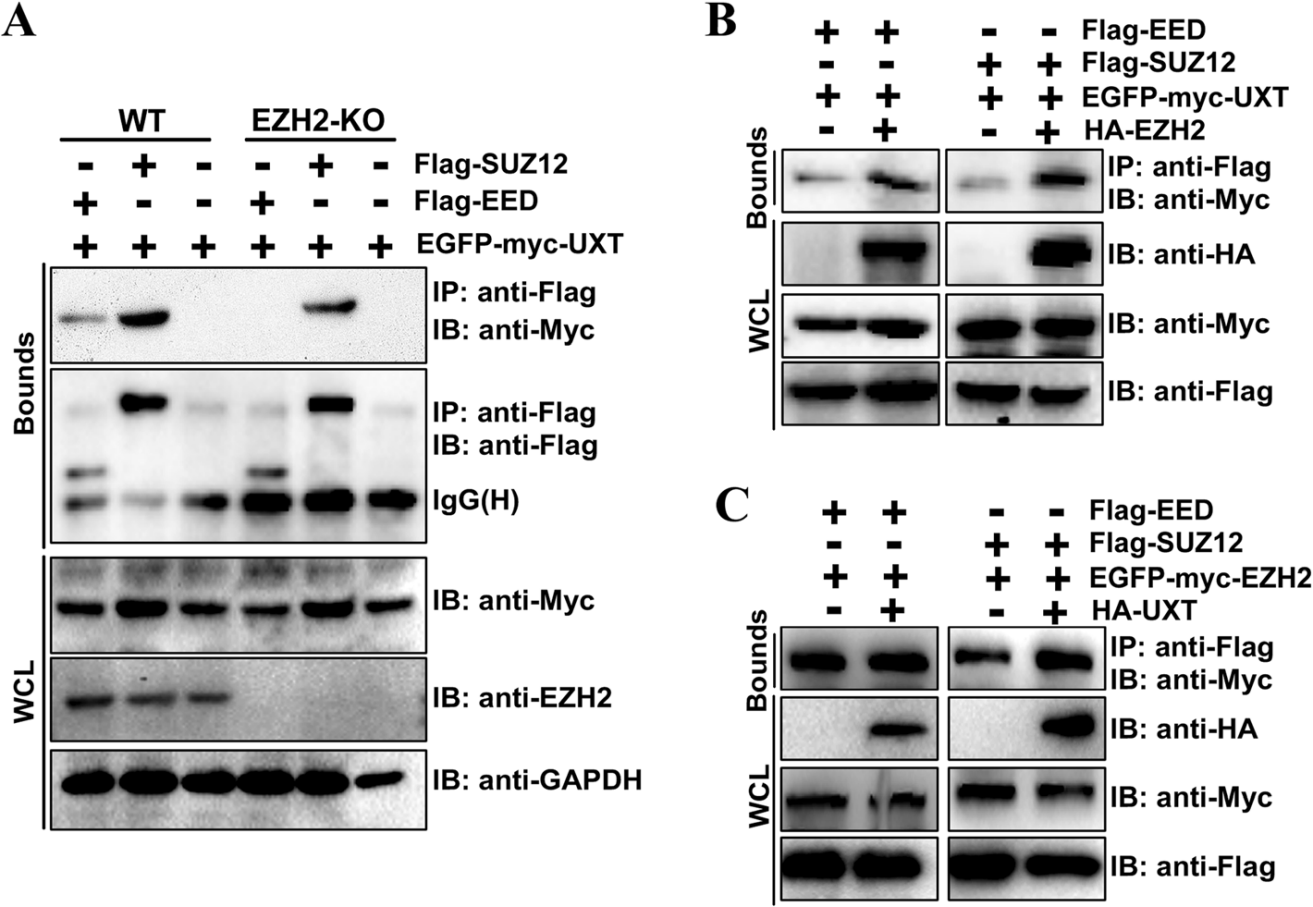
UXT promotes EZH2-SUZ12 interaction. **a** UXT interacts with SUZ12, but not with EED, in EZH2-knockout cells. Cotransfection of EGFP-myc-UXT into 786-O or EZH2-knockout 786-O cells was performed, together with Flag-SUZ12, Flag-EED, or an empty control plasmid psi-Flag, respectively. 48 h after transfection, the whole cell lysate was extracted for co-immunoprecipitation with anti-Flag, followed by probing with anti-Myc. **b** EZH2 facilitates UXT binding with the other PRC2 components SUZ12 and EED. 293 T cells were co-transfected with the indicated combinations of plasmids. Cell lysates were precipitated with anti-Flag antibody and immunoblotted with anti-Myc antibody. **c** UXT binds to EZH2 and promotes the interaction between EZH2 and SUZ12, but has no effect on EZH2-EED interaction. 293 T cells were co-transfected with the indicated combinations of plasmids. Cell lysates were precipitated with anti-Flag antibody and immunoblotted with anti-Myc antibody

EZH2 is classically associated with PRC2 complex, and both PRC2 components, including SUZ12 and EED. These three PRC2 components are required for its HMTase activity. Thus, we examined whether UXT interacts with SUZ12 and EED. Co-immunoprecipitation assays of Flag-EED or Flag-SUZ12 with EGFP-myc-UXT in 786-O cells showed that UXT was coprecipitated efficiently with both EED and SUZ12 (Fig. 4a; lanes 1–3), which suggested that UXT is associated with the PRC2 complex. However, knockout of EZH2 led to disruption of EED binding to UXT but did not disrupt the binding to SUZ12 (Fig. 4a; lanes 4 and 5). These results suggested that UXT are directly associated with EZH2 and SUZ12, and EZH2 mediates the interaction between UXT and EED. Furthermore, there was weaker binding between SUZ12 and UXT in EZH2 knockout cells (Fig. 4a; lanes 5 versus 2), comparing to wild-type cells, which suggested that EZH2 facilitates UXT binding to SUZ12. Indeed, the overexpression of EZH2 resulted in the promotion of EGFP-myc-UXT binding to EED or SUZ12 in 293 T cells (Fig. 4b). Further investigation showed that overexpression of UXT had no effect on interaction between EZH2 and EED (Fig. 4c; lanes 1 and 2). However, ectopically expressed UXT was found to strengthen EZH2-SUZ12 interaction (Fig. 4c; lanes 3 versus 4). Taken together, these data support the hypothesis that UXT may promote EZH2 HMTase activity by contributing the binding between EZH2 and SUZ12.

### UXT promotes EZH2 HMTase activity

**Fig. 5:**
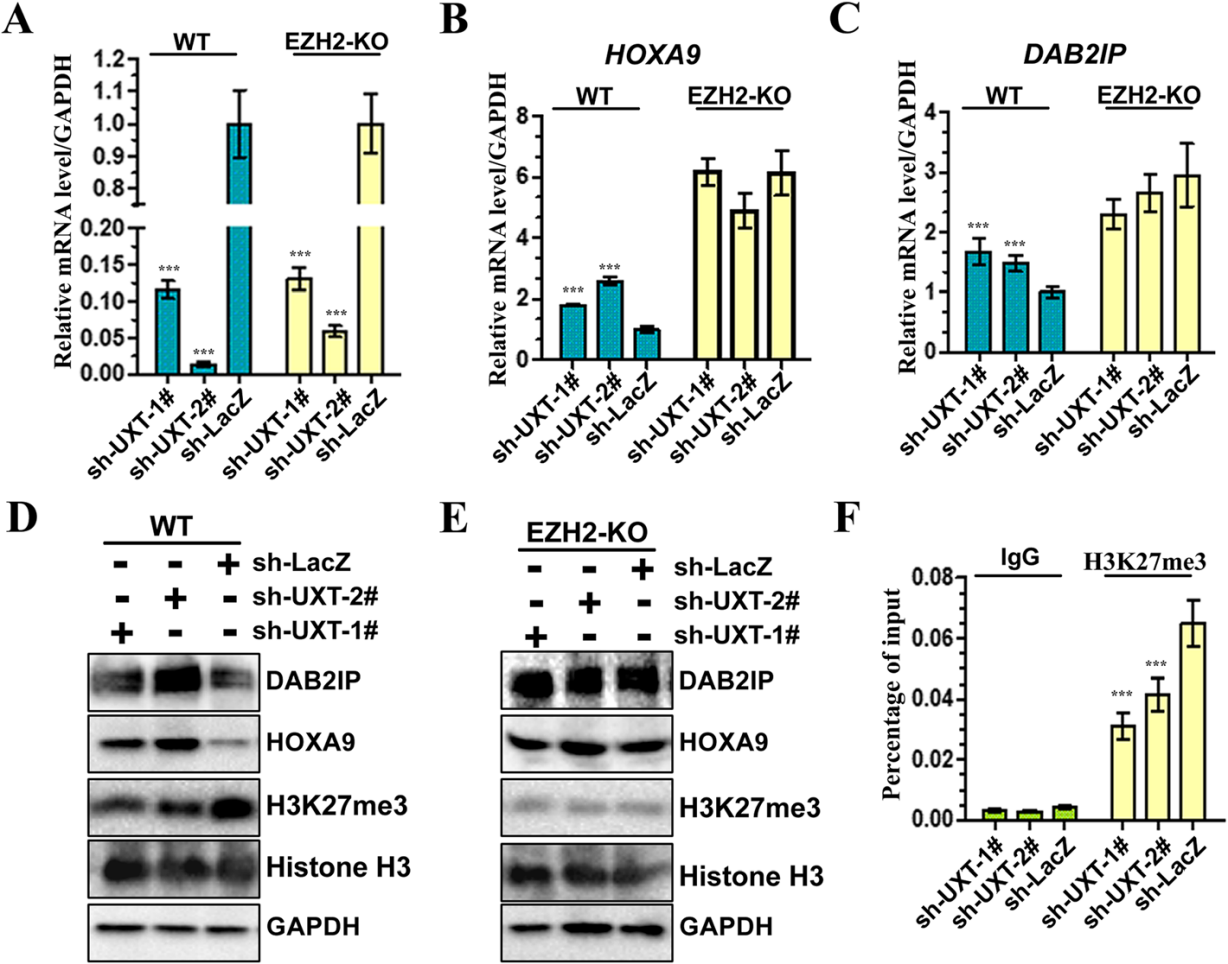
UXT promotes EZH2 HMTase activity. **a** Real-time PCR analysis of UXT mRNA showed efficient UXT knockdown by shRNA expression in 786-O and EZH2-knockout 786-O cells. **b**, **c** Real-time quantitative PCR shows that UXT knockdown promotes the expression of endogenous EZH2 target genes (*HOXA9* and *DAB2IP*) in EZH2 WT cells, but not in EZH2-knockout cells. Data are plotted as the mean ± SD of three independent experiments. **d**, **e** Western blot analysis shows upregulation of *DAB2IP* and *HOXA9* and downregulation of histone H3K27me3 in EZH2 WT 786-O cell lines (D), but not in EZH2-knockout cells(E). Total histone H3 and GAPDH were shown as control. **f** ChIP–qPCR analysis of H3K27me3 enrichment on *HOXA9* gene promoter in UXT knockdown cells. UXT positive regulates H3K27me3 levels at PRC2 target loci in 786-O cells

Given that EZH2 is histone H3 lysine 27 (H3K27) methyltransferase, which is associated with transcriptional repression, we sought to investigate the role of UXT in EZH2’s transcriptional repressor function. We stably inhibited UXT in both EZH2-WT and EZH2-KO 786-O cells. sh-UXT-1# and sh-UXT-2# incorporated cells were found to significantly reduced expression levels of UXT, compared with control cells (Fig. 5a). Next, we evaluated the expressions of EZH2-targets, including HOXA9 and DAB2IP, at both mRNA and protein levels by RT-qPCR and Western Blot. We found that HOXA9 and DAB2IP were upregulated by shRNA-mediated UXT knockdown in the EZH2-WT cells (Fig. 5b-c) but not in the EZH2-KO cells (Fig. 5b-c), which suggested that UXT depletion can promote the transcription of HOXA9 and DAB2IP through EZH2. Consistent with these results, knockdown of UXT in the EZH2-WT cells inhibited the EZH2 histone methyltransferase activity, indicated by the decrease of H3K27 methylation and increase of protein levels of HOXA9 and DAB2IP (Fig. 5d). In contrast, UXT depletion didn’t result in increase of expression levels of HOXA9 and DAB2IP in the EZH2-KO cells (Fig. 5e). In addition, by using quantitative chromatin immunoprecipitation (qChIP) assays, lower levels of H3K27 trimethylation(H3K27me3)on the HOXA9 promoter was detected in UXT knockdown cells comparing to the control cells (Fig. 5f). Taken together, these results suggest that UXT promotes formation of PRC2 and results in promotion of its HMTase activity.

### UXT is a renal cancer oncogene

**Fig. 6:**
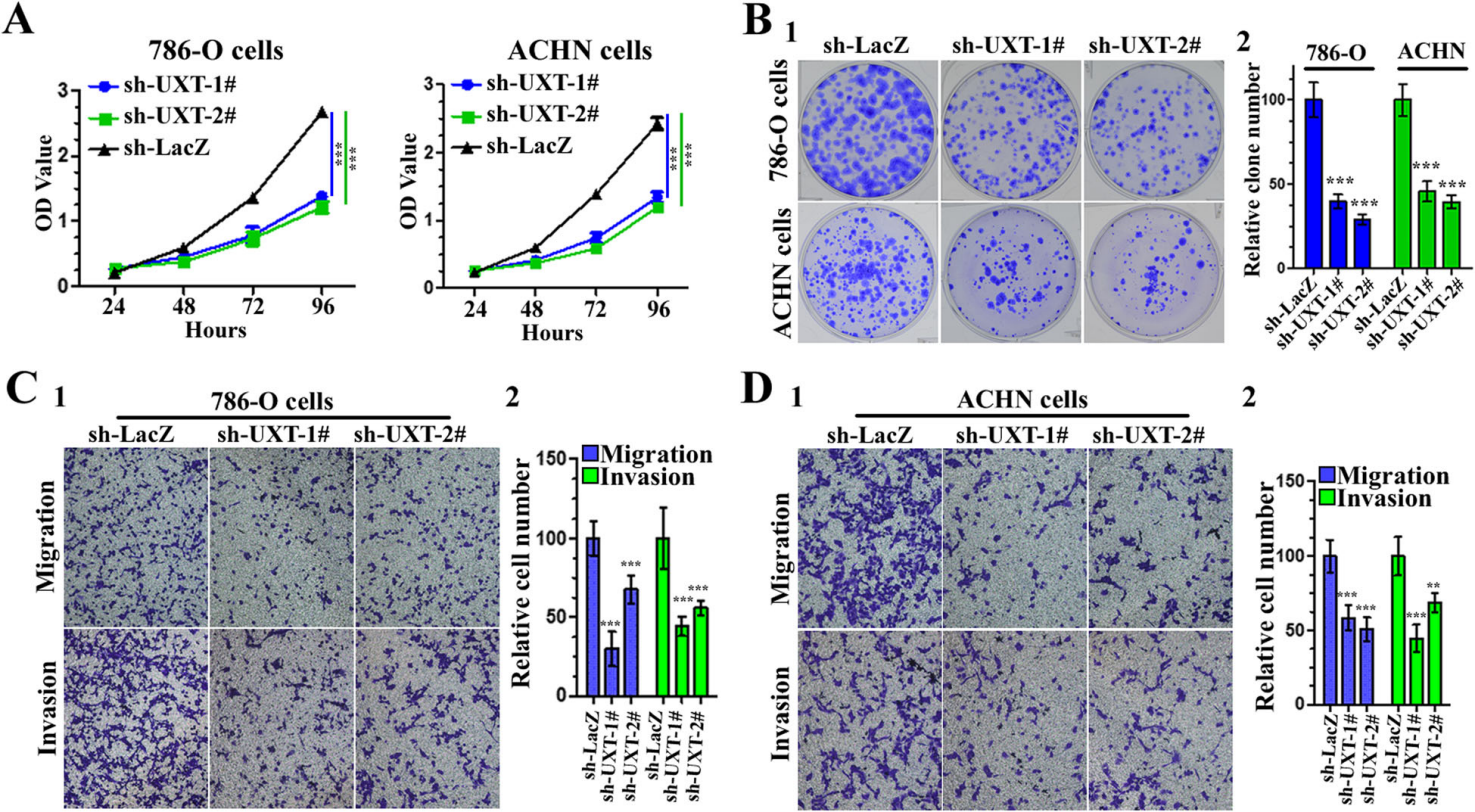
UXT is required for the cell proliferation, migration and invasion of renal cancer cells. **a** The cell viability of ACHN and 786-O cells expressing sh-LacZ or sh-UXT was determined by CCK8 assays at indicated time points. Data presented are means±SD from three independent experiments. **b** Colony formation assay in 786-O and ACHN cells. Relative colony formation units of sh-UXT- or sh-LacZ–transfected stable ACHN and 786-O cells (1). The number of colonies was quantified (2). **c**, **d** Migration and invasion assay in 786-O (**c**) and ACHN (**d**) cells. Representative photographs were taken at × 200 magnification (1). The number of migrated and invaded cells was quantified (2)

Since both UXT and EZH2 have been implicated in tumorigenesis, we next investigated whether UXT might regulate renal cancer tumorigenesis through modulating EZH2 HMTase activity. We and other groups have demonstrated that EZH2 function as an oncogene in ccRCC [30, 36–38]. However, the role of UXT in ccRCC is still under exploration. We first examined the tumorigenicity of UXT in renal cancer cells. As expected, UXT depletion inhibited cell proliferation and anchorage-dependent growth in ACHN and 786-O cells, as judged by CCK8 assays and colony formation (Fig. 6a-b). Secondly, knockdown of UXT inhibited migration and invasion of ACHN and 786-O cells (Fig. 6c-d). Together, these findings indicate that UXT is a potential candidate proto-oncogene in ccRCC.

**Fig. 7:**
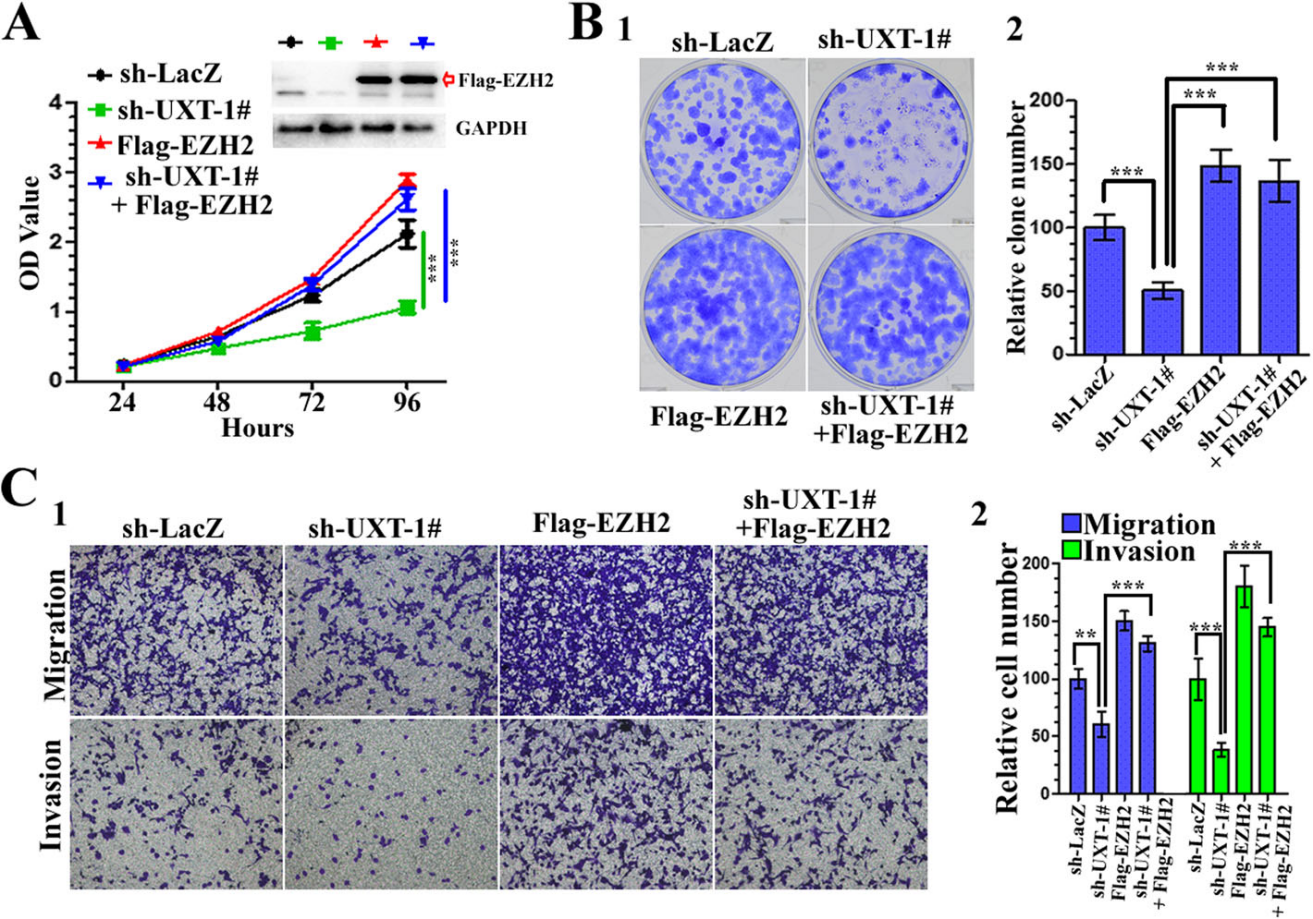
UXT regulates human renal cancer cell proliferation, migration and invasion via EZH2. **a** 786-O cells were infected with different combinations of lentivirus as indicated. 72 h after infection, CCK8 assay was utilized to quantify cell viability at each time point. Data are plotted as the mean ± SD of 3 independent experiments. Western blot analysis of the cells was performed to evaluate the expression of EZH2. GAPDH is used as an internal control. **b** After infection as (**a**), after infection, colony formation assay was performed to estimate the anchorage-independent growth ability of 786-O cells. Images were taken at 2 weeks after 2 weeks (1). The number of colonies was quantified (2). **c** After infection as (**a**), cells were used for Transwell assays. Representative photographs were taken at × 200 magnification (1). Number of migrated and invaded cells was quantified in 4 random images from each treatment group. Results are the mean ± SD from 3 independent experiments plotted as percent (%) migrating and invading cells relative to sh-LacZ treatment

Next, we tested whether exogenous EZH2 could overturn the suppressive effects of UXT knockdown on the tumorigenicity. We found that effects of UXT depletion on proliferation, migration, and invasion were reversed by concomitant EZH2 overexpression, which suggested that the effects of UXT on cell migration and invasion are mediated, at least in part, through EZH2 (Fig. 7). This finding supports the hypothesis that UXT promotes tumor progression (at least partially) through EZH2.

### UXT is increased in ccRCC patients and correlates negatively with survival

**Fig. 8:**
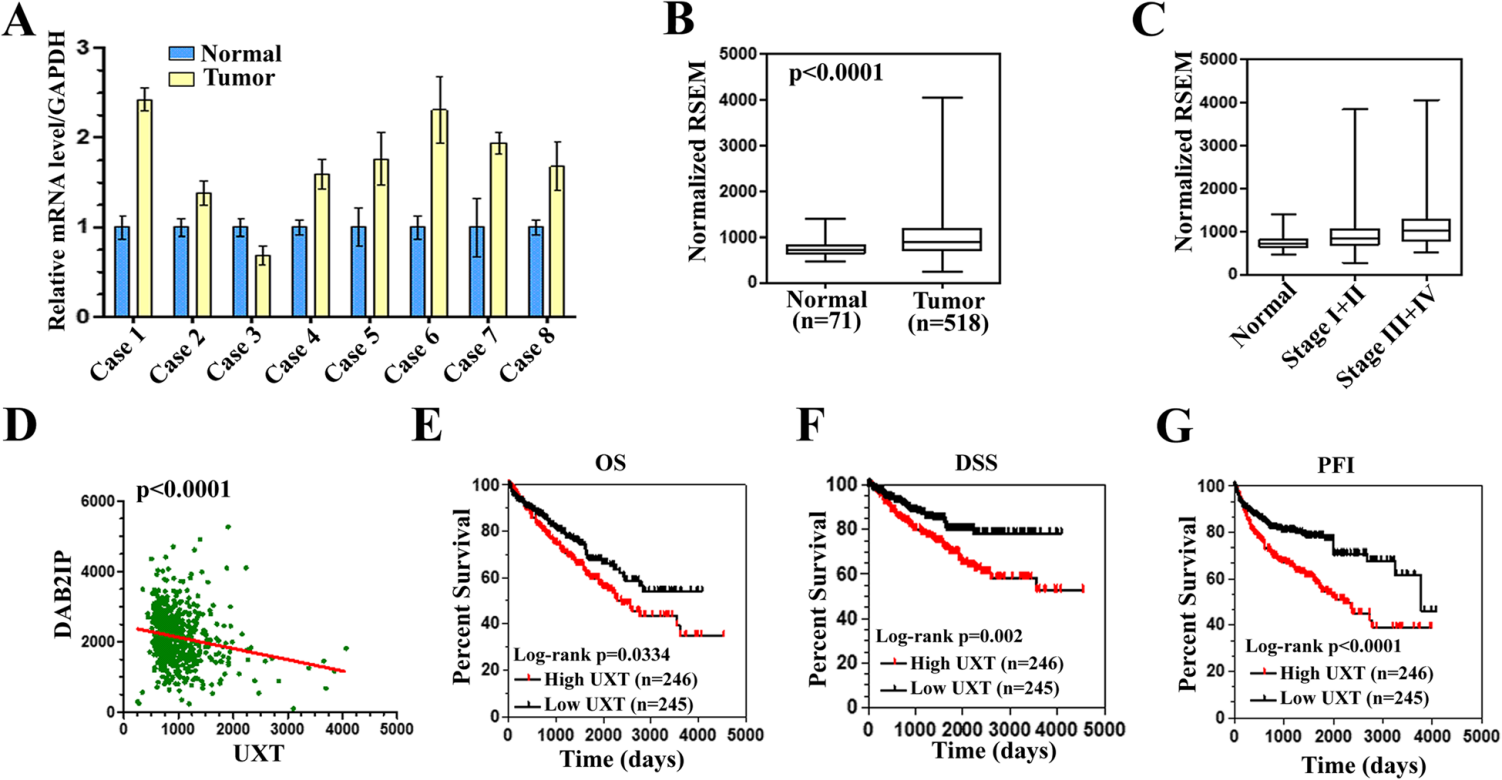
High expression of UXT is associated with advanced TNM stage and poor prognosis in patients with ccRCC. **a** Real-time quantitative PCR showed that UXT expression is increased in ccRCC patient tissue. **b** UXT mRNA levels in ccRCC patients downloaded from TCGA archive. Differential expression between ccRCC and normal tissues was calculated by using the downloaded RSEM (RNA-Seq by Expectation Maximization) values. **c** UXT is highly expressed in ccRCC patients with advanced TNM stage (III/IV stage). **d** UXT expression is negatively correlated with DAB2IP (*P* < 0.0001, r2 = 0.03686). **e**-**g** Clinical survival outcomes of ccRCC patients with high UXT expression (*N* = 246) or low UXT expression (*n* = 245). OS (**e**), DSS (**f**), and PFI (**g**). High expression of UXT is associated with poor prognosis in patients with ccRCC

To further investigate the role of UXT as a pro-oncogene, we examined its expression and clinical relevance in patients with ccRCC. First, qPCR analysis was performed to detect the mRNA level of UXT in 8 paired renal cancer samples and adjacent normal tissues. It was found that 7 of 8 tumors collected from renal cancer specimens have a significantly increased UXT level compared with corresponding normal controls (Fig. 8a). We next analyzed the expression levels of UXT in 518 tumors and 71 adjacent normal tissues of ccRCC from The Cancer Genome Atlas (TCGA) archive, and found that tumors have a significantly increased UXT level compared with adjacent normal tissues (Fig. 8b). In addition, the UXT expression was significantly positive correlated with TNM stage (*P* = 0.0003) (Table 1). UXT was increased 1.26-fold (P = 0.0003) in low-stage (Stages I and II) and 1.48-fold (*P* < 0.0001) in advanced-stage (Stages III and IV) tumors, demonstrating that UXT expression is associated with advanced tumor stages (Fig. 8c). Interestingly, UXT expression negatively correlated with DAB2IP (P < 0.0001, r^2^ = 0.03686, Spearman correlation) (Fig. 8d), corroborating our observation that UXT promotes the EZH2 HMTase activity, which inhibits the transcription of DAB2IP. Importantly, increased UXT expression is probably associated with advanced distant metastasis and poor survival in patients with ccRCC (Table 1). A higher expression of UXT was closely associated with poor overall survival (OS) (Fig. 8e), disease-specific survival (DSS) (Fig. 8f), and progression-free interval (PFI) (Fig. 8g) in patients with ccRCC. These results indicate that UXT might be recognized as a prognostic factor for cancer survival. Taken together, these results validate the mechanistic link between increased UXT expression and ccRCC progression, which supported the conclusion that UXT is a renal cancer oncogene which affects the progression and survival of ccRCC patients.

**Table 1:** Clinical characteristics and outcome of 491 ccRCC patients downloaded from TCGA according to UXT gene expression status

Characteristic	Total (*n* = 491)	UXT		P
		High (n = 246)	Low (n = 245)	
Gender				*P* = 0.0006
Male	313	175	138	
Female	178	71	107	
Age				*P* = 0.0643
≤60	242	111	131	
>60	249	135	114	
Mean (SD)	60.8 (12.1)	61.7 (12.1)	60.0 (12.0)	
Grade				*P* = 0.0007
G1-G2	223	92	128	
G3-G4	267	153	114	
GX	3	1	3	
TNM Stage				P = 0.0003
I-II	293	127	166	
III-IV	198	119	79	
T Stage				*P* = 0.0023
T1-T2	210	139	171	
T3-T4	181	107	74	
Lymph node metastasis				*P* = 0.6509
N0	220	108	112	
N1	14	6	8	
NX	258	132	126	
Distant metastasis				*P* = 0.0431
M0	385	187	198	
M1	75	46	29	
MX	31	13	18	
Vital status				*P* = 0.0009
Dead	163	99	64	
Alive	328	147	181	

Two-tailed chi-square test was done with the SPSS software program to determine the statistical significance of the level of expression of UXT in different Clinical characteristics, *P* < 0.05 was considered significant

## Discussion

In the present study, we present a newly identified mechanism for UXT in ccRCC, where UXT regulates tumor progression by regulating PRC2 activity in renal cancer cells. By using yeast two-hybrid to identify protein partners of UXT, we uncovered interactions between UXT and EZH2 in PRC2 complex. Specifically, we suggest that UXT promotes EZH2 HMTase activity by promoting the formation of PRC2 complex. In a previous study [35], it was proposed that the UXT interacts with the EZH1-SUZ12 complex to regulate the transcription of NF-κB target genes, and demonstrated that UXT neither binds EZH2 nor regulates its HMTase activity in HCT116 cells. In this regard, we recognized that these results contradict ours and examined this issue from several perspectives. We demonstrated that UXT can bind to EZH2 by various assays, including yeast two-hybrid, GST pull down, and co-immunoprecipitation. Since protein-protein interactions (PPIs) are dynamic process, and sometimes, are specific to the cell type and cell cycle phase [39, 40], it is possible that UXT-EZH2 interaction is context-dependent or/and cell-type specific. Further studies are necessary to elucidate the reason for this discrepancy. In addition, we found that multiple domain of EZH2 can interact with UXT. Since UXT contains one α domain and shows homology to prefoldin which functions as a co-chaperone and plays important role in protein folding [35], which suggests that UXT may regulate the protein folding of PRC2 complex.

UXT plays a significant role in regulating the progression of various tumors, including prostate and breast cancer [14, 15, 17, 24–27] and is classically known as both coactivator and corepressor protein that participates in the regulation of several transcription factors by direct interaction. UXT can interact with the N terminus of AR and facilitate receptor dependent transcriptional activation, which contributes to its role in AR-dependent prostate tumorigenesis [14, 25]. It has also been reported that UXT functions primarily as an AR corepressor and represses transcription of AR downstream genes [26]. UXT also modulates estrogen receptor-α activity by interacting with LOX-PP in breast cancer cells [24]. In addition, UXT also interacts with many other well-known tumor-associated transcription factors, such as NF-κB, EVI1, GATA4, FOG2, NKX2.5, and Foxp3 [15–17, 41], which suggests that UXT may regulate tumorigenesis though multiple signaling pathways. The mechanism by which UXT affects tumorigenesis remains to be defined.

PRC2 complex catalyzes the mono-, di-, and trimethylation of lysine 27 of histone H3, which plays an important role in the regulation of multiple biological processes, including tumor initiation and progression [42, 43]. EZH2 is the catalytic subunit of PRC2, which has been shown to be dysregulated in various different cancer types, including ccRCC [30, 36–38]. Mechanistically, EZH2 methylates H3K27 to promote transcriptional silencing of many tumor suppressor genes [44]. In addition to histone, many other non-histone proteins also methylated by EZH2 [45–49], and the interaction between EZH2 and these non-histone substrates plays important role in the development and progression of a variety of cancers. Interestingly, EZH2 serves as a coactivator of AR through direct interaction to facilitate its oncogenic function in cells of castration-resistant prostate cancer [49]. Furthermore, EZH2 also functions either as a novel positive or negative regulator of NF-κB depending on the ER status [50]. Given that UXT also functions as a transcriptional co-regulator of AR [14, 26], NF-κB [15, 18], and ER [24], it will be very interesting to determine whether UXT and EZH2 synergistically regulate the activity of these transcription factors.

In this study, knockdown of UXT demonstrated that this protein plays an essential role in ccRCC. More importantly, we found that EZH2 was sufficient to rescue the growth defects following UXT inhibition. Thus, our results support a model that UXT regulates the formation of PRC2 complex and plays an important role in repression of PRC2 target genes, therefore, promotes ccRCC progression. Consistently, UXT is upregulated in renal clear cell carcinomas and highly negative correlates with DAB2IP expression. Furthermore, we analyzed the data of over 500 ccRCC patients retrieved from TCGA archive, and found that the expression level of UXT is clearly correlated with the grade, stage and metastasis of tumors. Interestingly, we also found that UXT was highly expressed among male ccRCC patients. As a co-regulator of AR, UXT may also be up-regulated by AR in a feedback manner, which helps to explain the reason for higher expression level of UXT in male over female patient with ccRCC. Further elucidation of the mechanism of UXT in the promotion of EZH2 activity may provide new therapeutic targets for ccRCC.

## Conclusion

In summary, we discovered a novel UXT/PRC2 complex that represses the expression of EZH2 target genes. And the UXT acts as a renal cancer oncogene that affects the progression and survival of ccRCC patients.

## Additional files

Additional file 1: **Table S1** The primers used in the study. (DOC 44 kb)

Additional file 2: **Table S2** The potential UXT-interacting proteins identified by a yeast two-hybrid assay. (DOCX 19 kb)

## Data Availability

The datasets used and analyzed during the current study are available from the corresponding author on reasonable request.

## Abbreviations

ccRCC: Clear cell renal cell carcinoma
EED: Embryonic ectoderm development
EZH2: Enhancer of zeste homolog 2
H3K27: Histone H3 lysine 27
H3K27me3: Histone H3 lysine 27 trimethylation
HMTase: Histone methyltransferase
PRC2: Polycomb repressive complex 2
RCC: Renal cell carcinoma
SUZ12: Suppressor of zeste 12 homolog
UXT: Ubiquitous expressed transcript;
