# Supplementary material for: Ubiquitous expressed transcript promotes tumorigenesis by acting as a positive modulator of the polycomb repressive complex 2 in clear cell renal cell carcinoma
Source: BMC Cancer. 2019 Sep 3;19:874. doi: 10.1186/s12885-019-6069-3 (PMC6724258; doi:10.1186/s12885-019-6069-3)
Supplement: Supplementary file 1 — Table S1 The primers used in the study. (DOC 44 kb) [file 12885_2019_6069_MOESM1_ESM.doc]

**Additional file 1 Table S1. The primers used in the study**

Sites for restriction enzymes are underlined.

| **Primer name** | **Primer sequence (5′-3′)** | **PCR** |
| --- | --- | --- |
| **Primers for making constructs** | | |
| UXT-5'  UXT-3' | CGCGGATCCATGGTCTTCCCCCTCCCCACT  CCGCTCGAGTCAATGGTGAGGCTTCTCTGGGA | 94 °C, 30 s; 58 °C, 30 s; 72 °C, 30 s; 35 cycles |
| EZH2-5'  EZH2-3' | CGCGGATCCATGGGCCAGACTGGGAAGAAAT  CCGCTCGAGTCAAGGGATTTCCATTTCTCTTTC | 94 °C, 30 s; 58 °C, 30 s; 72 °C, 90 s; 35 cycles |
| EZH2(1-170)-3’  EZH2(170-340)-5’  EZH2(170-340)-3’  EZH2(341-559)-5’  EZH2(341-559)-3’  EZH2(560-751)-5’ | CCGCTCGAGTCAAATTTCATCATTTATAAACCCAC  CGCGGATCCATTTTTGTGGAGTTGGTGAATG  CCGCTCGAGAGCAAACTCCTTTGCTCCCT  CGCGGATCCGCTGCTCTCACCGCTGAG  CCGCTCGAGGAACTACATTGACAAAACTTTTC  CGCGGATCCAGAGTGTCAAAACCGCTTTCC | 94 °C, 30 s; 58 °C, 30 s; 72 °C, 90 s; 35 cycles |
| H1-F  H1-R | CCATCGATAATTCGAACGCTGACGTCAT  AAAAGCGGCCGCGGATCCGAGTGGTCTCATACAGA | 94 °C, 30 s; 58 °C, 30 s; 72 °C, 30 s; 35 cycles |
| sh-UXT-1#-5’  sh-UXT-1#-3’ | GATCCGGTATATGAGCAGCTGGCCAACTCGAGTTGGCCAGCTGCTCATATACCTTTTTT  GGCCAAAAAAGGTATATGAGCAGCTGGCCAACTCGAGTTGGCCAGCTGCTCATATACCG | |
| sh-UXT-2#-5’  sh-UXT-2#-3’ | GATCCGCTCTCAAGTTCATTGATCGCTCGAGCGATCAATGAACTTGAGAGCTTTTTT  GGCCAAAAAAGCTCTCAAGTTCATTGATCGCTCGAGCGATCAATGAACTTGAGAGCG | |
| sh-LacZ-5’  sh-LacZ-3’ | GATCCGTGACCAGCGAATACCTGTCTCGAGACAGGTATTCGCTGGTCACTTTTTT  GGCCAAAAAAGTGACCAGCGAATACCTGTCTCGAGACAGGTATTCGCTGGTCACG | |
| **Primers for real-time quantitative PCR analysis** | | |
| GAPDH | F: TGGTCACCAGGGCTGCTTTTA  R: CATCGCCCCACTTGATTTTG | 94°C, 30s; 60°C, 30s; 72°C, 30s; 40 cycles |
| UXT | F: AGGTGGATTTGGGCTGTAAC  R: GATATTCATGGAGTCCTTGGT | 94°C, 30s; 60°C, 30s; 72°C, 30s; 40 cycles |
| HOXA9 | F: TTGGAGGAAATGAATGCTGA  R: TGGTCAGTAGGCCTTGAGGT | 94°C, 30s; 60°C, 30s; 72°C, 30s; 40 cycles |
| DAB2IP | F: TGGACGATGTGCTCTATGCC  R: GGATGGTGATGGTTTGGTAG | 94°C, 30s; 60°C, 30s; 72°C, 30s; 40 cycles |
| **Primers for ChIP-qPCR analysis** | | |
| HOXA9  (promoter) | F: TCGCCAACCAAACACAACAGTC  R: AAAGGGATCGTGCCGCTCTAC | 95°C, 15s; 60°C, 15s; 72°C, 45s; 40 cycles |
