# Supplementary material for: Ubiquitous expressed transcript promotes tumorigenesis by acting as a positive modulator of the polycomb repressive complex 2 in clear cell renal cell carcinoma
Source: BMC Cancer. 2019 Sep 3;19:874. doi: 10.1186/s12885-019-6069-3 (PMC6724258; doi:10.1186/s12885-019-6069-3)
Supplement: Supplementary file 2 — Table S2 The potential UXT-interacting proteins identified by a yeast two-hybrid assay. (DOCX 19 kb) [file 12885_2019_6069_MOESM2_ESM.docx]

**Additional file 2Table S2. The potential UXT-interacting proteins identified by a yeast two-hybrid assay**

| **Clone ID** | **Gene Symbol** | **Description** | **Peptides range** |
| --- | --- | --- | --- |
| 1 | WDR85 | WD repeat domain 85 | 326-452 |
| 2 | LPCAT4 | lysophosphatidylcholine acyltransferase 4 | 425-524 |
| 3 | RPIA | ribose 5-phosphate isomerase A | 144-311 |
| 4 | UXT | ubiquitously-expressed transcript isoform 2 | 2-157 |
| 5 | PSMB4 | proteasome beta 4 subunit | 1-264 |
| 6 | RPSA | ribosomal protein SA | 73-283 |
| 7 | ACLY | ATP citrate lyase isoform 1 | 476-705 |
| 8 | EZH2 | enhancer of zeste 2 isoform a | 569-751 |
| 9 | DDAH2 | dimethylarginine dimethylaminohydrolase 2 | 25-285 |
| 10 | RPSA | ribosomal protein SA | 111-295 |
| 11 | FBLN5 | fibulin 5 precursor | 285-448 |
| 12 | NEUROD1 | neurogenic differentiation 1 | 131-356 |
| 13 | EIF3F | eukaryotic translation initiation factor 3, subunit 5 epsilon | 63-301 |
| 14 | DCK | deoxycytidine kinase | 160-260 |
| 15 | NDUFB9 | NADH dehydrogenase (ubiquinone) 1 beta subcomplex, 9 | 35-179 |
| 16 | NDUFB9 | NADH dehydrogenase (ubiquinone) 1 beta subcomplex, 9 | 35-179 |
| 17 | NDUFB9 | NADH dehydrogenase (ubiquinone) 1 beta subcomplex, 9 | 35-179 |
| 18 | DCK | deoxycytidine kinase | 160-260 |
| 19 | NDUFB9 | NADH dehydrogenase (ubiquinone) 1 beta subcomplex, 9 | 35-179 |
